# High blood pressure and associated risk factors as indicator of preclinical hypertension in rural West Africa: A focus on children and adolescents in The Gambia: Erratum

**DOI:** 10.1097/MD.0000000000007402

**Published:** 2017-06-23

In the article, “High blood pressure and associated risk factors as indicator of preclinical hypertension in rural West Africa: A focus on children and adolescents in The Gambia”,^[1]^ which appeared in Volume 96, Issue 13 of *Medicine*, the licensing has been changed to “This license lets others distribute, remix, tweak, and build upon your work, even commercially, as long as they credit you for the original creation. This is the most accommodating of licenses offered. Recommended for maximum dissemination and use of licensed materials.”
